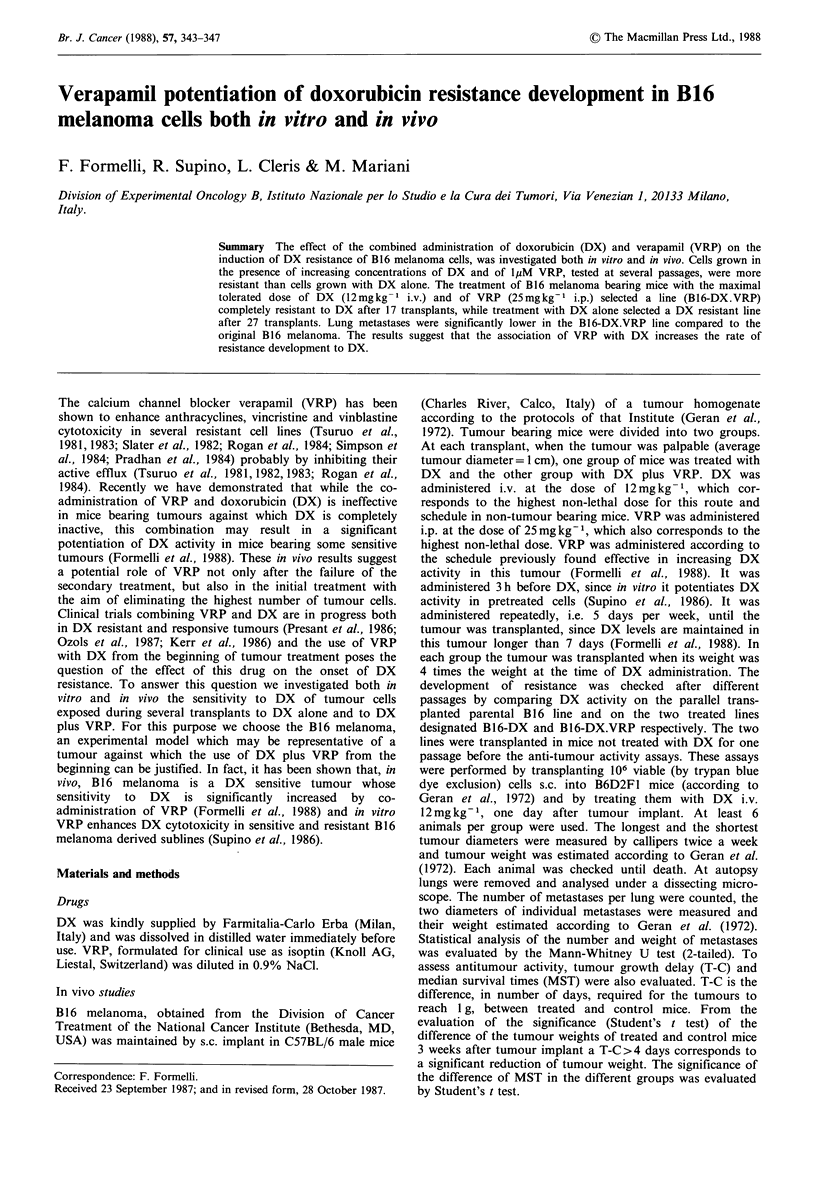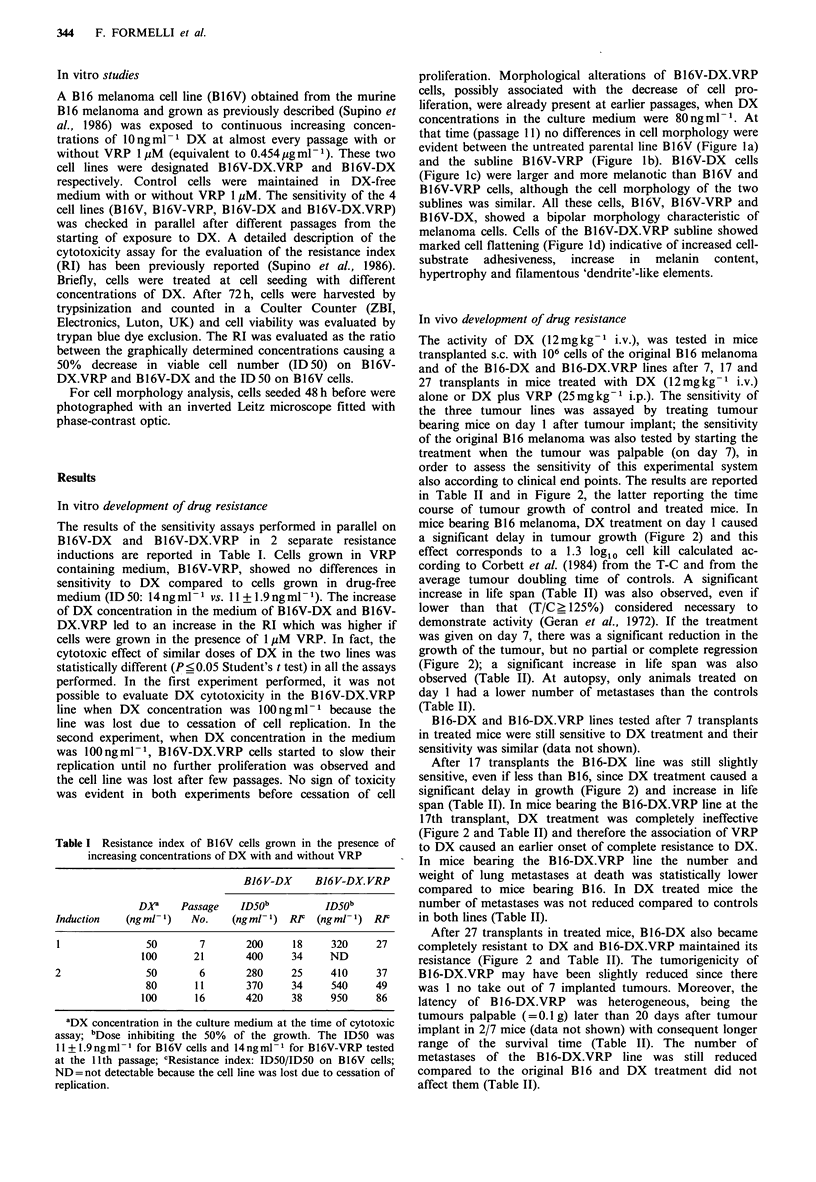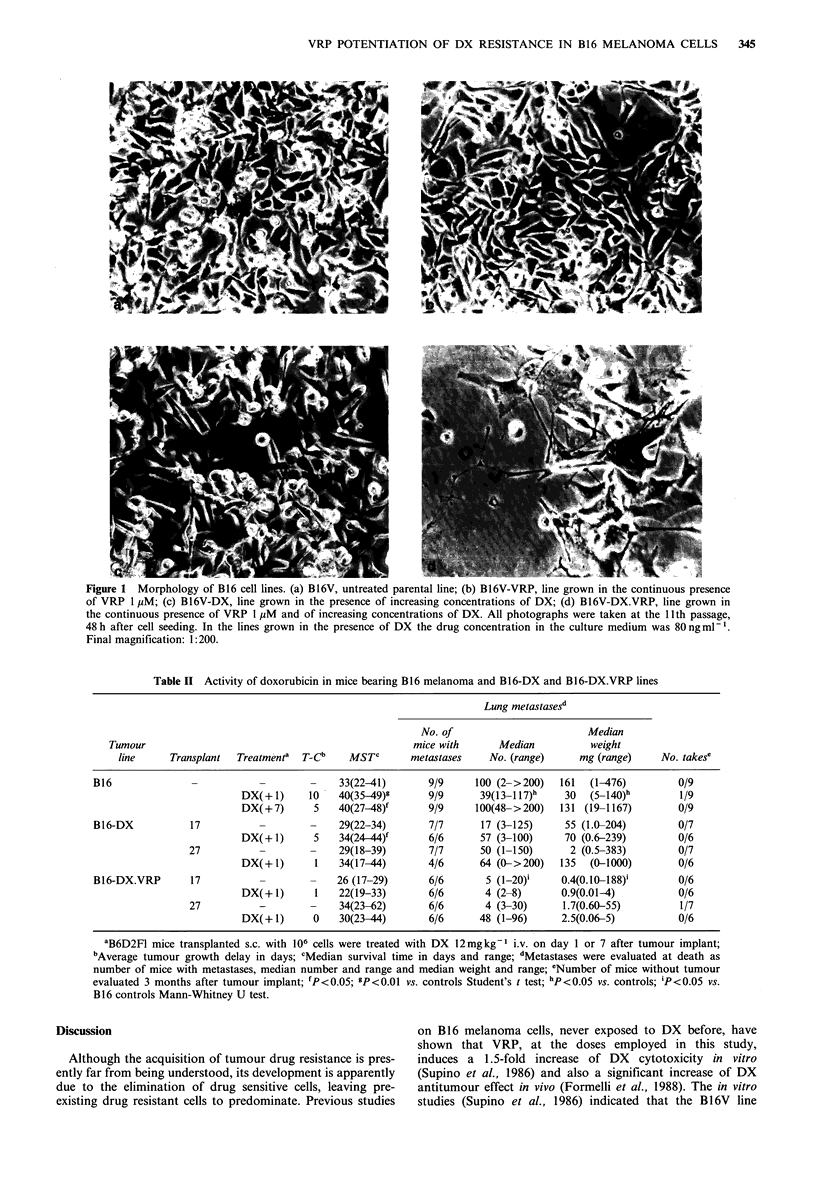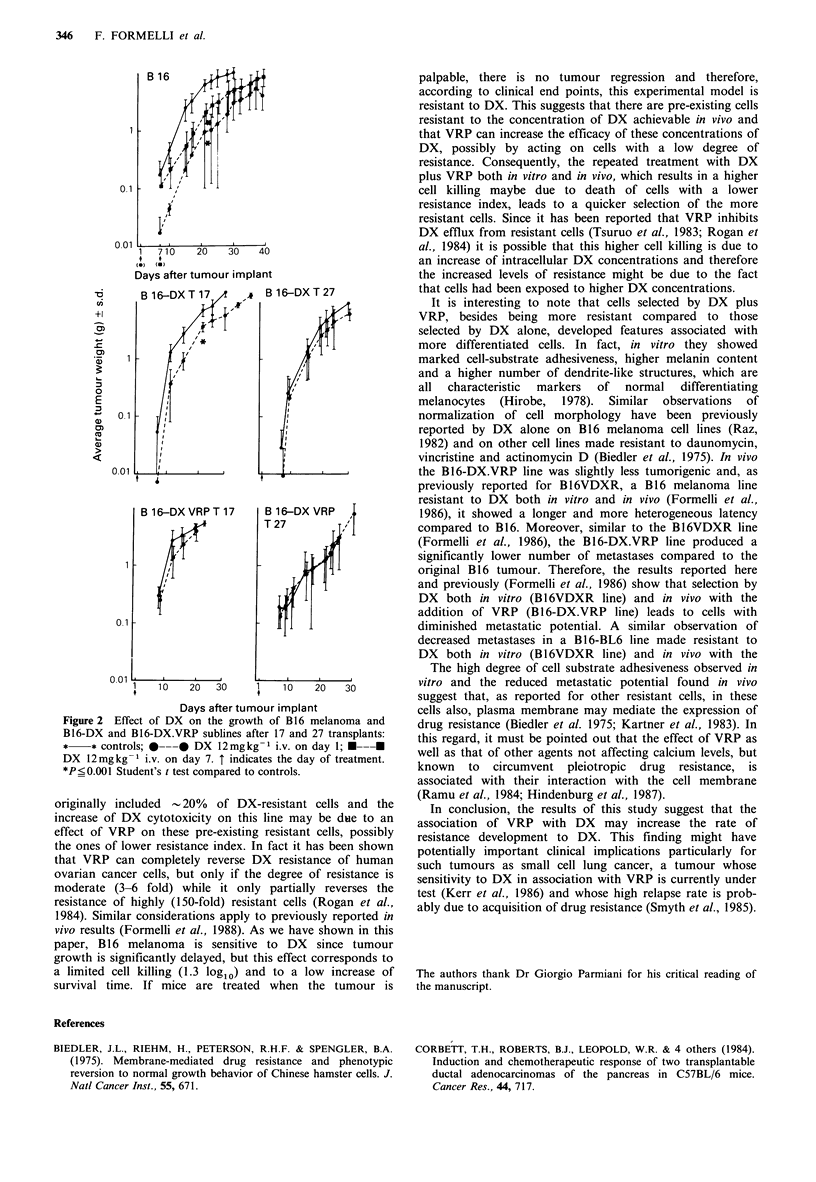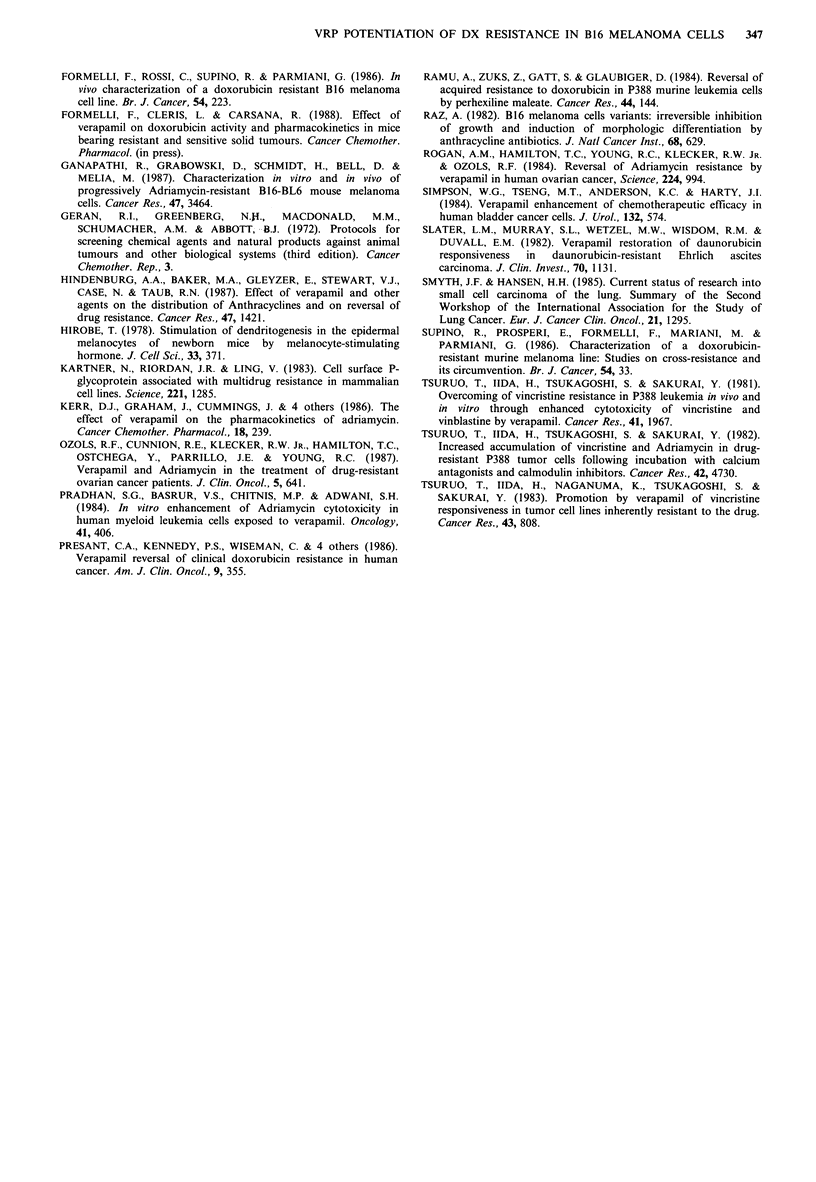# Verapamil potentiation of doxorubicin resistance development in B16 melanoma cells both in vitro and in vivo.

**DOI:** 10.1038/bjc.1988.79

**Published:** 1988-04

**Authors:** F. Formelli, R. Supino, L. Cleris, M. Mariani

**Affiliations:** Division of Experimental Oncology B, Istituto Nazionale per lo Studio e la Cura dei Tumori, Milano, Italy.

## Abstract

**Images:**


Br. J. Cancer (1988), 57, 343–347 © The Macmillan Press Ltd., 1988

### Verapamil potentiation of doxorubicin resistance development in B16 melanoma cells both *in vitro* and *in vivo*

F. Formelli, R. Supino, L. Cleris & M. Mariani

*Division of Experimental Oncology B, Istituto Nazionale per lo Studio e la Cura dei Tumori, Via Venezian 1, 20133 Milano, Italy.*

**Summary** The effect of the combined administration of doxorubicin (DX) and verapamil (VRP) on the induction of DX resistance of B16 melanoma cells, was investigated both *in vitro* and *in vivo*. Cells grown in the presence of increasing concentrations of DX and of 1$\mu$M VRP, tested at several passages, were more resistant than cells grown with DX alone. The treatment of B16 melanoma bearing mice with the maximal tolerated dose of DX (12 mg kg$^{-1}$ i.v.) and of VRP (25 mg kg$^{-1}$ i.p.) selected a line (B16-DX.VRP) completely resistant to DX after 17 transplants, while treatment with DX alone selected a DX resistant line after 27 transplants. Lung metastases were significantly lower in the B16-DX.VRP line compared to the original B16 melanoma. The results suggest that the association of VRP with DX increases the rate of resistance development to DX.

The calcium channel blocker verapamil (VRP) has been shown to enhance anthracyclines, vincristine and vinblastine cytotoxicity in several resistant cell lines (Tsuruo *et al.*, 1981, 1983; Slater *et al.*, 1982; Rogan *et al.*, 1984; Simpson *et al.*, 1984; Pradhan *et al.*, 1984) probably by inhibiting their active efflux (Tsuruo *et al.*, 1981, 1982, 1983; Rogan *et al.*, 1984). Recently we have demonstrated that while the co-administration of VRP and doxorubicin (DX) is ineffective in mice bearing tumours against which DX is completely inactive, this combination may result in a significant potentiation of DX activity in mice bearing some sensitive tumours (Formelli *et al.*, 1988). These *in vivo* results suggest a potential role of VRP not only after the failure of the secondary treatment, but also in the initial treatment with the aim of eliminating the highest number of tumour cells. Clinical trials combining VRP and DX are in progress both in DX resistant and responsive tumours (Presant *et al.*, 1986; Ozols *et al.*, 1987; Kerr *et al.*, 1986) and the use of VRP with DX from the beginning of tumour treatment poses the question of the effect of this drug on the onset of DX resistance. To answer this question we investigated both *in vitro* and *in vivo* the sensitivity to DX of tumour cells exposed during several transplants to DX alone and to DX plus VRP. For this purpose we choose the B16 melanoma, an experimental model which may be representative of a tumour against which the use of DX plus VRP from the beginning can be justified. In fact, it has been shown that, *in vivo*, B16 melanoma is a DX sensitive tumour whose sensitivity to DX is significantly increased by co-administration of VRP (Formelli *et al.*, 1988) and *in vitro* VRP enhances DX cytotoxicity in sensitive and resistant B16 melanoma derived sublines (Supino *et al.*, 1986).

### Materials and methods

### *Drugs*

DX was kindly supplied by Farmitalia-Carlo Erba (Milan, Italy) and was dissolved in distilled water immediately before use. VRP, formulated for clinical use as isoptin (Knoll AG, Liestal, Switzerland) was diluted in 0.9% NaCl.

### In vivo *studies*

B16 melanoma, obtained from the Division of Cancer Treatment of the National Cancer Institute (Bethesda, MD, USA) was maintained by s.c. implant in C57BL/6 male mice (Charles River, Calco, Italy) of a tumour homogenate according to the protocols of that Institute (Geran *et al.*, 1972). Tumour bearing mice were divided into two groups. At each transplant, when the tumour was palpable (average tumour diameter = 1 cm), one group of mice was treated with DX and the other group with DX plus VRP. DX was administered i.v. at the dose of 12 mg kg$^{-1}$, which corresponds to the highest non-lethal dose for this route and schedule in non-tumour bearing mice. VRP was administered i.p. at the dose of 25 mg kg$^{-1}$, which also corresponds to the highest non-lethal dose. VRP was administered according to the schedule previously found effective in increasing DX activity in this tumour (Formelli *et al.*, 1988). It was administered 3 h before DX, since *in vitro* it potentiates DX activity in pretreated cells (Supino *et al.*, 1986). It was administered repeatedly, i.e. 5 days per week, until the tumour was transplanted, since DX levels are maintained in this tumour longer than 7 days (Formelli *et al.*, 1988). In each group the tumour was transplanted when its weight was 4 times the weight at the time of DX administration. The development of resistance was checked after different passages by comparing DX activity on the parallel transplanted parental B16 line and on the two treated lines designated B16-DX and B16-DX.VRP respectively. The two lines were transplanted in mice not treated with DX for one passage before the anti-tumour activity assays. These assays were performed by transplanting $10^6$ viable (by trypan blue dye exclusion) cells s.c. into B6D2F1 mice (according to Geran *et al.*, 1972) and by treating them with DX i.v. 12 mg kg$^{-1}$, one day after tumour implant. At least 6 animals per group were used. The longest and the shortest tumour diameters were measured by callipers twice a week and tumour weight was estimated according to Geran *et al.* (1972). Each animal was checked until death. At autopsy lungs were removed and analysed under a dissecting microscope. The number of metastases per lung were counted, the two diameters of individual metastases were measured and their weight estimated according to Geran *et al.* (1972). Statistical analysis of the number and weight of metastases was evaluated by the Mann-Whitney U test (2-tailed). To assess antitumour activity, tumour growth delay (T-C) and median survival times (MST) were also evaluated. T-C is the difference, in number of days, required for the tumours to reach 1 g, between treated and control mice. From the evaluation of the significance (Student's *t* test) of the difference of the tumour weights of treated and control mice 3 weeks after tumour implant a T-C > 4 days corresponds to a significant reduction of tumour weight. The significance of the difference of MST in the different groups was evaluated by Student's *t* test.

Correspondence: F. Formelli.
Received 23 September 1987; and in revised form, 28 October 1987.

### In vitro *studies*

A B16 melanoma cell line (B16V) obtained from the murine B16 melanoma and grown as previously described (Supino *et al.*, 1986) was exposed to continuous increasing concentrations of 10 ng ml$^{-1}$ DX at almost every passage with or without VRP 1 $\mu$M (equivalent to 0.454 $\mu$g ml$^{-1}$). These two cell lines were designated B16V-DX.VRP and B16V-DX respectively. Control cells were maintained in DX-free medium with or without VRP 1 $\mu$M. The sensitivity of the 4 cell lines (B16V, B16V-VRP, B16V-DX and B16V-DX.VRP) was checked in parallel after different passages from the starting of exposure to DX. A detailed description of the cytotoxicity assay for the evaluation of the resistance index (RI) has been previously reported (Supino *et al.*, 1986). Briefly, cells were treated at cell seeding with different concentrations of DX. After 72 h, cells were harvested by trypsinization and counted in a Coulter Counter (ZBI, Electronics, Luton, UK) and cell viability was evaluated by trypan blue dye exclusion. The RI was evaluated as the ratio between the graphically determined concentrations causing a 50% decrease in viable cell number (ID 50) on B16V-DX.VRP and B16V-DX and the ID 50 on B16V cells.

For cell morphology analysis, cells seeded 48 h before were photographed with an inverted Leitz microscope fitted with phase-contrast optic.

### Results

### In vitro *development of drug resistance*

The results of the sensitivity assays performed in parallel on B16V-DX and B16V-DX.VRP in 2 separate resistance inductions are reported in Table I. Cells grown in VRP containing medium, B16V-VRP, showed no differences in sensitivity to DX compared to cells grown in drug-free medium (ID 50: 14 ng ml$^{-1}$ *vs.* 11 $\pm$ 1.9 ng ml$^{-1}$). The increase of DX concentration in the medium of B16V-DX and B16V-DX.VRP led to an increase in the RI which was higher if cells were grown in the presence of 1 $\mu$M VRP. In fact, the cytotoxic effect of similar doses of DX in the two lines was statistically different ($P \leq 0.05$ Student's *t* test) in all the assays performed. In the first experiment performed, it was not possible to evaluate DX cytotoxicity in the B16V-DX.VRP line when DX concentration was 100 ng ml$^{-1}$ because the line was lost due to cessation of cell replication. In the second experiment, when DX concentration in the medium was 100 ng ml$^{-1}$, B16V-DX.VRP cells started to slow their replication until no further proliferation was observed and the cell line was lost after few passages. No sign of toxicity was evident in both experiments before cessation of cell proliferation. Morphological alterations of B16V-DX.VRP cells, possibly associated with the decrease of cell proliferation, were already present at earlier passages, when DX concentrations in the culture medium were 80 ng ml$^{-1}$. At that time (passage 11) no differences in cell morphology were evident between the untreated parental line B16V (Figure 1a) and the subline B16V-VRP (Figure 1b). B16V-DX cells (Figure 1c) were larger and more melanotic than B16V and B16V-VRP cells, although the cell morphology of the two sublines was similar. All these cells, B16V, B16V-VRP and B16V-DX, showed a bipolar morphology characteristic of melanoma cells. Cells of the B16V-DX.VRP subline showed marked cell flattening (Figure 1d) indicative of increased cell-substrate adhesiveness, increase in melanin content, hypertrophy and filamentous 'dendrite'-like elements.

**Table I** Resistance index of B16V cells grown in the presence of increasing concentrations of DX with and without VRP

| | | | *B16V-DX* | | *B16V-DX.VRP* | |
|---|---|---|---|---|---|---|
| *Induction* | *DX*[a] *(ng ml$^{-1}$)* | *Passage No.* | *ID50*[b] *(ng ml$^{-1}$)* | *RI*[c] | *ID50*[b] *(ng ml$^{-1}$)* | *RI*[c] |
| 1 | 50 | 7 | 200 | 18 | 320 | 27 |
| | 100 | 21 | 400 | 34 | ND | |
| 2 | 50 | 6 | 280 | 25 | 410 | 37 |
| | 80 | 11 | 370 | 34 | 540 | 49 |
| | 100 | 16 | 420 | 38 | 950 | 86 |

[a]DX concentration in the culture medium at the time of cytotoxic assay; [b]Dose inhibiting the 50% of the growth. The ID50 was 11 $\pm$ 1.9 ng ml$^{-1}$ for B16V cells and 14 ng ml$^{-1}$ for B16V-VRP tested at the 11th passage; [c]Resistance index: ID50/ID50 on B16V cells; ND = not detectable because the cell line was lost due to cessation of replication.

### In vivo *development of drug resistance*

The activity of DX (12 mg kg$^{-1}$ i.v.), was tested in mice transplanted s.c. with $10^6$ cells of the original B16 melanoma and of the B16-DX and B16-DX.VRP lines after 7, 17 and 27 transplants in mice treated with DX (12 mg kg$^{-1}$ i.v.) alone or DX plus VRP (25 mg kg$^{-1}$ i.p.). The sensitivity of the three tumour lines was assayed by treating tumour bearing mice on day 1 after tumour implant; the sensitivity of the original B16 melanoma was also tested by starting the treatment when the tumour was palpable (on day 7), in order to assess the sensitivity of this experimental system also according to clinical end points. The results are reported in Table II and in Figure 2, the latter reporting the time course of tumour growth of control and treated mice. In mice bearing B16 melanoma, DX treatment on day 1 caused a significant delay in tumour growth (Figure 2) and this effect corresponds to a 1.3 $\log_{10}$ cell kill calculated according to Corbett *et al.* (1984) from the T-C and from the average tumour doubling time of controls. A significant increase in life span (Table II) was also observed, even if lower than that (T/C $\geq$ 125%) considered necessary to demonstrate activity (Geran *et al.*, 1972). If the treatment was given on day 7, there was a significant reduction in the growth of the tumour, but no partial or complete regression (Figure 2); a significant increase in life span was also observed (Table II). At autopsy, only animals treated on day 1 had a lower number of metastases than the controls (Table II).

B16-DX and B16-DX.VRP lines tested after 7 transplants in treated mice were still sensitive to DX treatment and their sensitivity was similar (data not shown).

After 17 transplants the B16-DX line was still slightly sensitive, even if less than B16, since DX treatment caused a significant delay in growth (Figure 2) and increase in life span (Table II). In mice bearing the B16-DX.VRP line at the 17th transplant, DX treatment was completely ineffective (Figure 2 and Table II) and therefore the association of VRP to DX caused an earlier onset of complete resistance to DX. In mice bearing the B16-DX.VRP line the number and weight of lung metastases at death was statistically lower compared to mice bearing B16. In DX treated mice the number of metastases was not reduced compared to controls in both lines (Table II).

After 27 transplants in treated mice, B16-DX also became completely resistant to DX and B16-DX.VRP maintained its resistance (Figure 2 and Table II). The tumorigenicity of B16-DX.VRP may have been slightly reduced since there was 1 no take out of 7 implanted tumours. Moreover, the latency of B16-DX.VRP was heterogeneous, being the tumours palpable (= 0.1 g) later than 20 days after tumour implant in 2/7 mice (data not shown) with consequent longer range of the survival time (Table II). The number of metastases of the B16-DX.VRP line was still reduced compared to the original B16 and DX treatment did not affect them (Table II).

**Figure 1** Morphology of B16 cell lines. (a) B16V, untreated parental line; (b) B16V-VRP, line grown in the continuous presence of VRP 1 $\mu$M; (c) B16V-DX, line grown in the presence of increasing concentrations of DX; (d) B16V-DX.VRP, line grown in the continuous presence of VRP 1 $\mu$M and of increasing concentrations of DX. All photographs were taken at the 11th passage, 48 h after cell seeding. In the lines grown in the presence of DX the drug concentration in the culture medium was 80 ng ml$^{-1}$. Final magnification: 1:200.

**Table II** Activity of doxorubicin in mice bearing B16 melanoma and B16-DX and B16-DX.VRP lines

| *Tumour line* | *Transplant* | *Treatment*[a] | *T-C*[b] | *MST*[c] | *Lung metastases*[d] *No. of mice with metastases* | *Median No. (range)* | *Median weight mg (range)* | *No. takes*[e] |
|---|---|---|---|---|---|---|---|---|
| B16 | – | – | – | 33(22–41) | 9/9 | 100 (2–>200) | 161 (1–476) | 0/9 |
| | | DX(+1) | 10 | 40(35–49)[g] | 9/9 | 39(13–117)[h] | 30 (5–140)[h] | 1/9 |
| | | DX(+7) | 5 | 40(27–48)[f] | 9/9 | 100(48–>200) | 131 (19–1167) | 0/9 |
| B16-DX | 17 | – | – | 29(22–34) | 7/7 | 17 (3–125) | 55 (1.0–204) | 0/7 |
| | | DX(+1) | 5 | 34(24–44)[f] | 6/6 | 57 (3–100) | 70 (0.6–239) | 0/6 |
| | 27 | – | – | 29(18–39) | 7/7 | 50 (1–150) | 2 (0.5–383) | 0/7 |
| | | DX(+1) | 1 | 34(17–44) | 4/6 | 64 (0–>200) | 135 (0–1000) | 0/6 |
| B16-DX.VRP | 17 | – | – | 26 (17–29) | 6/6 | 5 (1–20)[i] | 0.4(0.10–188)[i] | 0/6 |
| | | DX(+1) | 1 | 22(19–33) | 6/6 | 4 (2–8) | 0.9(0.01–4) | 0/6 |
| | 27 | – | – | 34(23–62) | 6/6 | 4 (3–30) | 1.7(0.60–55) | 1/7 |
| | | DX(+1) | 0 | 30(23–44) | 6/6 | 48 (1–96) | 2.5(0.06–5) | 0/6 |

[a]B6D2Fl mice transplanted s.c. with $10^6$ cells were treated with DX 12 mg kg$^{-1}$ i.v. on day 1 or 7 after tumour implant; [b]Average tumour growth delay in days; [c]Median survival time in days and range; [d]Metastases were evaluated at death as number of mice with metastases, median number and range and median weight and range; [e]Number of mice without tumour evaluated 3 months after tumour implant; [f]$P<0.05$; [g]$P<0.01$ *vs.* controls Student's *t* test; [h]$P<0.05$ *vs.* controls; [i]$P<0.05$ *vs.* B16 controls Mann-Whitney U test.

### Discussion

Although the acquisition of tumour drug resistance is presently far from being understood, its development is apparently due to the elimination of drug sensitive cells, leaving pre-existing drug resistant cells to predominate. Previous studies on B16 melanoma cells, never exposed to DX before, have shown that VRP, at the doses employed in this study, induces a 1.5-fold increase of DX cytotoxicity *in vitro* (Supino *et al.*, 1986) and also a significant increase of DX antitumour effect *in vivo* (Formelli *et al.*, 1988). The *in vitro* studies (Supino *et al.*, 1986) indicated that the B16V line

**Figure 2** Effect of DX on the growth of B16 melanoma and B16-DX and B16-DX.VRP sublines after 17 and 27 transplants: *——* controls; ●---● DX 12 mg kg$^{-1}$ i.v. on day 1; ■---■ DX 12 mg kg$^{-1}$ i.v. on day 7. ↑ indicates the day of treatment. *$P \leq 0.001$ Student's $t$ test compared to controls.

originally included ~20% of DX-resistant cells and the increase of DX cytotoxicity on this line may be due to an effect of VRP on these pre-existing resistant cells, possibly the ones of lower resistance index. In fact it has been shown that VRP can completely reverse DX resistance of human ovarian cancer cells, but only if the degree of resistance is moderate (3–6 fold) while it only partially reverses the resistance of highly (150-fold) resistant cells (Rogan *et al.*, 1984). Similar considerations apply to previously reported *in vivo* results (Formelli *et al.*, 1988). As we have shown in this paper, B16 melanoma is sensitive to DX since tumour growth is significantly delayed, but this effect corresponds to a limited cell killing (1.3 $\log_{10}$) and to a low increase of survival time. If mice are treated when the tumour is palpable, there is no tumour regression and therefore, according to clinical end points, this experimental model is resistant to DX. This suggests that there are pre-existing cells resistant to the concentration of DX achievable *in vivo* and that VRP can increase the efficacy of these concentrations of DX, possibly by acting on cells with a low degree of resistance. Consequently, the repeated treatment with DX plus VRP both *in vitro* and *in vivo*, which results in a higher cell killing maybe due to death of cells with a lower resistance index, leads to a quicker selection of the more resistant cells. Since it has been reported that VRP inhibits DX efflux from resistant cells (Tsuruo *et al.*, 1983; Rogan *et al.*, 1984) it is possible that this higher cell killing is due to an increase of intracellular DX concentrations and therefore the increased levels of resistance might be due to the fact that cells had been exposed to higher DX concentrations.

It is interesting to note that cells selected by DX plus VRP, besides being more resistant compared to those selected by DX alone, developed features associated with more differentiated cells. In fact, *in vitro* they showed marked cell-substrate adhesiveness, higher melanin content and a higher number of dendrite-like structures, which are all characteristic markers of normal differentiating melanocytes (Hirobe, 1978). Similar observations of normalization of cell morphology have been previously reported by DX alone on B16 melanoma cell lines (Raz, 1982) and on other cell lines made resistant to daunomycin, vincristine and actinomycin D (Biedler *et al.*, 1975). *In vivo* the B16-DX.VRP line was slightly less tumorigenic and, as previously reported for B16VDXR, a B16 melanoma line resistant to DX both *in vitro* and *in vivo* (Formelli *et al.*, 1986), it showed a longer and more heterogeneous latency compared to B16. Moreover, similar to the B16VDXR line (Formelli *et al.*, 1986), the B16-DX.VRP line produced a significantly lower number of metastases compared to the original B16 tumour. Therefore, the results reported here and previously (Formelli *et al.*, 1986) show that selection by DX both *in vitro* (B16VDXR line) and *in vivo* with the addition of VRP (B16-DX.VRP line) leads to cells with diminished metastatic potential. A similar observation of decreased metastases in a B16-BL6 line made resistant to DX both *in vitro* (B16VDXR line) and *in vivo* with the

The high degree of cell substrate adhesiveness observed *in vitro* and the reduced metastatic potential found *in vivo* suggest that, as reported for other resistant cells, in these cells also, plasma membrane may mediate the expression of drug resistance (Biedler *et al.* 1975; Kartner *et al.*, 1983). In this regard, it must be pointed out that the effect of VRP as well as that of other agents not affecting calcium levels, but known to circumvent pleiotropic drug resistance, is associated with their interaction with the cell membrane (Ramu *et al.*, 1984; Hindenburg *et al.*, 1987).

In conclusion, the results of this study suggest that the association of VRP with DX may increase the rate of resistance development to DX. This finding might have potentially important clinical implications particularly for such tumours as small cell lung cancer, a tumour whose sensitivity to DX in association with VRP is currently under test (Kerr *et al.*, 1986) and whose high relapse rate is probably due to acquisition of drug resistance (Smyth *et al.*, 1985).

The authors thank Dr Giorgio Parmiani for his critical reading of the manuscript.

### References

BIEDLER, J.L., RIEHM, H., PETERSON, R.H.F. & SPENGLER, B.A. (1975). Membrane-mediated drug resistance and phenotypic reversion to normal growth behavior of Chinese hamster cells. *J. Natl Cancer Inst.*, **55**, 671.

CORBETT, T.H., ROBERTS, B.J., LEOPOLD, W.R. & 4 others (1984). Induction and chemotherapeutic response of two transplantable ductal adenocarcinomas of the pancreas in C57BL/6 mice. *Cancer Res.*, **44**, 717.

FORMELLI, F., ROSSI, C., SUPINO, R. & PARMIANI, G. (1986). *In vivo* characterization of a doxorubicin resistant B16 melanoma cell line. *Br. J. Cancer*, **54**, 223.

FORMELLI, F., CLERIS, L. & CARSANA, R. (1988). Effect of verapamil on doxorubicin activity and pharmacokinetics in mice bearing resistant and sensitive solid tumours. *Cancer Chemother. Pharmacol.* (in press).

GANAPATHI, R., GRABOWSKI, D., SCHMIDT, H., BELL, D. & MELIA, M. (1987). Characterization *in vitro* and *in vivo* of progressively Adriamycin-resistant B16-BL6 mouse melanoma cells. *Cancer Res.*, **47**, 3464.

GERAN, R.I., GREENBERG, N.H., MACDONALD, M.M., SCHUMACHER, A.M. & ABBOTT, B.J. (1972). Protocols for screening chemical agents and natural products against animal tumours and other biological systems (third edition). *Cancer Chemother. Rep.*, **3**.

HINDENBURG, A.A., BAKER, M.A., GLEYZER, E., STEWART, V.J., CASE, N. & TAUB, R.N. (1987). Effect of verapamil and other agents on the distribution of Anthracyclines and on reversal of drug resistance. *Cancer Res.*, **47**, 1421.

HIROBE, T. (1978). Stimulation of dendritogenesis in the epidermal melanocytes of newborn mice by melanocyte-stimulating hormone. *J. Cell Sci.*, **33**, 371.

KARTNER, N., RIORDAN, J.R. & LING, V. (1983). Cell surface P-glycoprotein associated with multidrug resistance in mammalian cell lines. *Science*, **221**, 1285.

KERR, D.J., GRAHAM, J., CUMMINGS, J. & 4 others (1986). The effect of verapamil on the pharmacokinetics of adriamycin. *Cancer Chemother. Pharmacol.*, **18**, 239.

OZOLS, R.F., CUNNION, R.E., KLECKER, R.W. JR., HAMILTON, T.C., OSTCHEGA, Y., PARRILLO, J.E. & YOUNG, R.C. (1987). Verapamil and Adriamycin in the treatment of drug-resistant ovarian cancer patients. *J. Clin. Oncol.*, **5**, 641.

PRADHAN, S.G., BASRUR, V.S., CHITNIS, M.P. & ADWANI, S.H. (1984). *In vitro* enhancement of Adriamycin cytotoxicity in human myeloid leukemia cells exposed to verapamil. *Oncology*, **41**, 406.

PRESANT, C.A., KENNEDY, P.S., WISEMAN, C. & 4 others (1986). Verapamil reversal of clinical doxorubicin resistance in human cancer. *Am. J. Clin. Oncol.*, **9**, 355.

RAMU, A., ZUKS, Z., GATT, S. & GLAUBIGER, D. (1984). Reversal of acquired resistance to doxorubicin in P388 murine leukemia cells by perhexiline maleate. *Cancer Res.*, **44**, 144.

RAZ, A. (1982). B16 melanoma cells variants: irreversible inhibition of growth and induction of morphologic differentiation by anthracycline antibiotics. *J. Natl Cancer Inst.*, **68**, 629.

ROGAN, A.M., HAMILTON, T.C., YOUNG, R.C., KLECKER, R.W. JR. & OZOLS, R.F. (1984). Reversal of Adriamycin resistance by verapamil in human ovarian cancer, *Science*, **224**, 994.

SIMPSON, W.G., TSENG, M.T., ANDERSON, K.C. & HARTY, J.I. (1984). Verapamil enhancement of chemotherapeutic efficacy in human bladder cancer cells. *J. Urol.*, **132**, 574.

SLATER, L.M., MURRAY, S.L., WETZEL, M.W., WISDOM, R.M. & DUVALL, E.M. (1982). Verapamil restoration of daunorubicin responsiveness in daunorubicin-resistant Ehrlich ascites carcinoma. *J. Clin. Invest.*, **70**, 1131.

SMYTH, J.F. & HANSEN, H.H. (1985). Current status of research into small cell carcinoma of the lung. Summary of the Second Workshop of the International Association for the Study of Lung Cancer. *Eur. J. Cancer Clin. Oncol.*, **21**, 1295.

SUPINO, R., PROSPERI, E., FORMELLI, F., MARIANI, M. & PARMIANI, G. (1986). Characterization of a doxorubicin-resistant murine melanoma line: Studies on cross-resistance and its circumvention. *Br. J. Cancer*, **54**, 33.

TSURUO, T., IIDA, H., TSUKAGOSHI, S. & SAKURAI, Y. (1981). Overcoming of vincristine resistance in P388 leukemia *in vivo* and *in vitro* through enhanced cytotoxicity of vincristine and vinblastine by verapamil. *Cancer Res.*, **41**, 1967.

TSURUO, T., IIDA, H., TSUKAGOSHI, S. & SAKURAI, Y. (1982). Increased accumulation of vincristine and Adriamycin in drug-resistant P388 tumor cells following incubation with calcium antagonists and calmodulin inhibitors. *Cancer Res.*, **42**, 4730.

TSURUO, T., IIDA, H., NAGANUMA, K., TSUKAGOSHI, S. & SAKURAI, Y. (1983). Promotion by verapamil of vincristine responsiveness in tumor cell lines inherently resistant to the drug. *Cancer Res.*, **43**, 808.