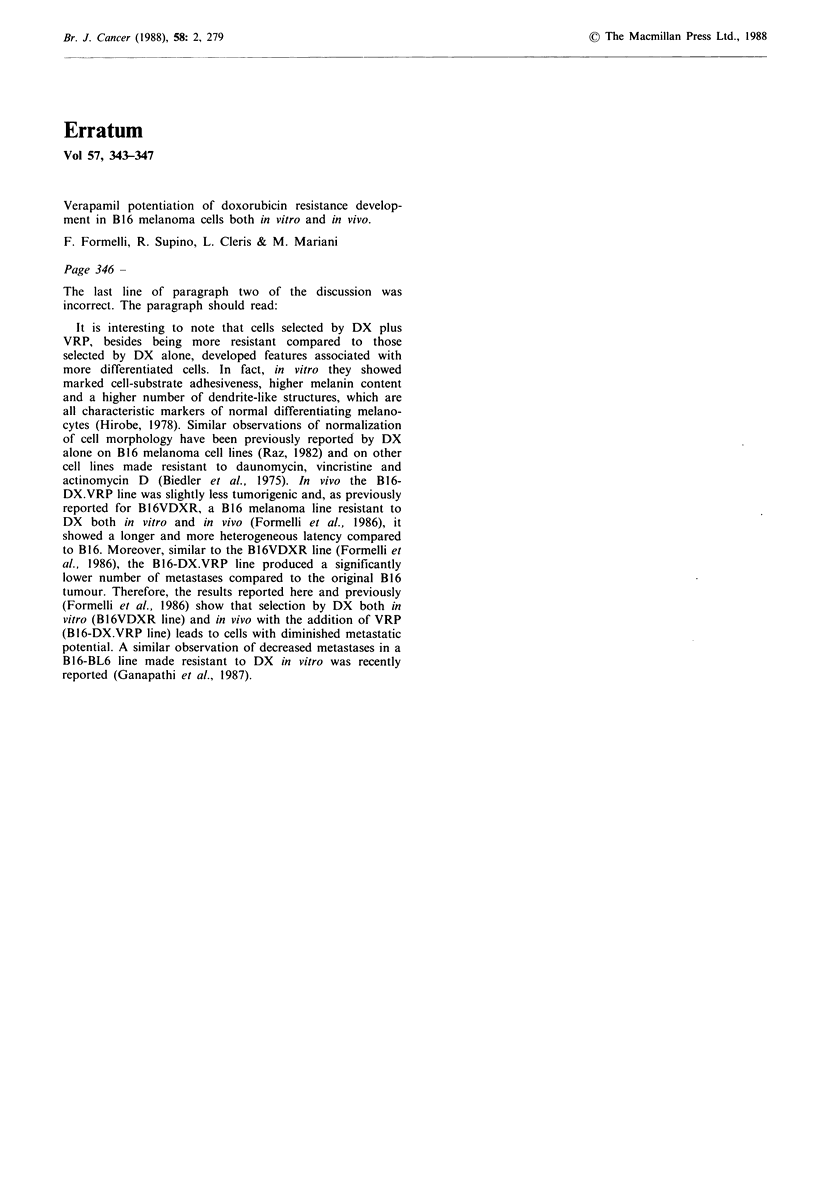# Erratum

**Published:** 1988-08

**Authors:** 


*Br. J. Cancer* (1988), **58**: 2, 279 © The Macmillan Press Ltd., 1988

### Erratum

**Vol 57, 343–347**

Verapamil potentiation of doxorubicin resistance development in B16 melanoma cells both *in vitro* and *in vivo*.

F. Formelli, R. Supino, L. Cleris & M. Mariani

*Page 346* –

The last line of paragraph two of the discussion was incorrect. The paragraph should read:

It is interesting to note that cells selected by DX plus VRP, besides being more resistant compared to those selected by DX alone, developed features associated with more differentiated cells. In fact, *in vitro* they showed marked cell-substrate adhesiveness, higher melanin content and a higher number of dendrite-like structures, which are all characteristic markers of normal differentiating melanocytes (Hirobe, 1978). Similar observations of normalization of cell morphology have been previously reported by DX alone on B16 melanoma cell lines (Raz, 1982) and on other cell lines made resistant to daunomycin, vincristine and actinomycin D (Biedler *et al.*, 1975). *In vivo* the B16-DX.VRP line was slightly less tumorigenic and, as previously reported for B16VDXR, a B16 melanoma line resistant to DX both *in vitro* and *in vivo* (Formelli *et al.*, 1986), it showed a longer and more heterogeneous latency compared to B16. Moreover, similar to the B16VDXR line (Formelli *et al.*, 1986), the B16-DX.VRP line produced a significantly lower number of metastases compared to the original B16 tumour. Therefore, the results reported here and previously (Formelli *et al.*, 1986) show that selection by DX both *in vitro* (B16VDXR line) and *in vivo* with the addition of VRP (B16-DX.VRP line) leads to cells with diminished metastatic potential. A similar observation of decreased metastases in a B16-BL6 line made resistant to DX *in vitro* was recently reported (Ganapathi *et al.*, 1987).